# Statin use decreases coagulation in users of vitamin K antagonists

**DOI:** 10.1007/s00228-016-2138-6

**Published:** 2016-10-05

**Authors:** Nienke van Rein, J. S. Biedermann, S. M. Bonafacio, M. J. H. A. Kruip, F. J. M. van der Meer, W. M. Lijfering

**Affiliations:** 1 Department of Thrombosis and Hemostasis, Leiden University Medical Center, Leiden, The Netherlands; 2 Einthoven Laboratory for Experimental Vascular Medicine, Leiden University Medical Center, Leiden, the Netherlands; 3 Department of Hematology, Erasmus University Medical Center, Rotterdam, The Netherlands; 4 Star-Medical Diagnostic Center, Rotterdam, The Netherlands; 5 Department of Clinical Epidemiology, Leiden University Medical Center, Leiden, The Netherlands

**Keywords:** Hydroxymethylglutaryl-CoA reductase inhibitors, Coumarins, Pharmacokinetics, Drug interactions, Pharmacology

## Abstract

**Purpose:**

The purpose of the study is to determine the immediate and long-term effect of statins on coagulation in patients treated with vitamin K antagonists (VKAs).

**Methods:**

We selected patients on VKAs of two Dutch anticoagulation clinics who initiated treatment with a statin between 2009 and 2013. Patients who initiated or stopped concomitant drugs that interact with VKAs or were hospitalised during follow-up were excluded. The VKA dosage (mg/day) after statin initiation was compared with the last VKA dosage before the statin was started. Immediate and long-term differences in VKA dosage (at 6 and 12 weeks) were calculated with a paired student *t* test.

**Results:**

Four hundred thirty-five phenprocoumon users (mean age 70 years, 60 % men) and 303 acenocoumarol users (mean age 69 years, 58 % men) were included. After start of statin use, the immediate phenprocoumon dosage was 0.02 mg/day (95 % CI, 0.00 to 0.03) lower. At 6 and 12 weeks, these phenprocoumon dosages were 0.03 (95 % CI, 0.01 to 0.05) and 0.07 mg/day (95 % CI, 0.04 to 0.09) lower as compared with the dosage before first statin use. In acenocoumarol users, VKA dosage was 0.04 mg/day (95%CI, 0.01 to 0.07) (immediate effect), 0.10 (95 % CI, 0.03 to 0.16) (at 6 weeks), and 0.11 mg/day (95 % CI, 0.04 to 0.18) (after 12 weeks) lower.

**Conclusions:**

Initiation of statin treatment was associated with an immediate and long-term minor although statistically significant decrease in VKA dosage in both phenprocoumon and acenocoumarol users, which suggests that statins may have anticoagulant properties.

## Introduction

Patients on vitamin K antagonists (VKAs) often have or develop arterial cardiovascular morbidity [1], for which they require cardiovascular drugs like statins [2]. Statins are competitive inhibitors of HMG-CoA reductase that reduce cholesterol biosynthesis [3], but may also reduce the risk of venous thrombosis [4, 5]. However, the anticoagulant properties of statins are not well defined and it is unclear how statins could lower the risk of venous thrombosis [5]. Currently, three randomised studies and one observational study have been conducted as to observe if statins have anticoagulant properties in VKA users [2, 6–8]. These studies showed conflicting results possibly due to the low number of participants enrolled or because (for the observational study) of residual confounding [2, 6–8]. In addition, it is unclear whether potential anticoagulant properties of statins in VKA users are due to drug-drug interactions with VKA or due to pharmacodynamic effects of the statins. To gain more insight into the effects of statins on coagulation in VKA users, we compared coagulation of patients on VKAs before and up till 12 weeks after starting statin therapy.

## Methods

### Study design, patient selection, and data collection

Patients’ characteristics and outcomes were collected from the computerised records of the anticoagulation clinic Leiden and the Star-Medical Diagnostic Center, Rotterdam. At these anticoagulation clinics, all patients are monitored at least every 6 weeks. At each visit, a standard questionnaire is performed regarding initiation of concomitant medications and planned procedures after which blood is drawn to determine the international normalised ratio (INR). Based on the INR, the VKA dosage until the next visit is set by a trained physician at the anticoagulation clinic.

All patients, who started treatment with VKAs (i.e. phenprocoumon or acenocoumarol) between January 2009 and December 2013, were screened. This time period was chosen as the coded registration of statin treatment in our databases started in 2009. Patients were included who started to use a statin within this period. Patients were excluded if they started to use inegy (combination of ezetimibe and simvastatin) because it is unknown to which degree both medications are attributable to a possible anticoagulant effect. Patients who were already using statins at baseline or started to use a statin within the first 2 months of VKA treatment were also excluded. Patients were excluded when they were hospitalised between the INR measurement before and after statin initiation because for example an acute myocardial infarction may affect coagulation and therefore change the outcome (INR and dosage of VKA). Patients were also excluded if they started or stopped any interacting medication with VKAs, according to the national list of medication interacting with VKA of Dutch Anticoagulation Clinics [9], during their individual observation period within this study. Neither informed consent nor approval by a medical ethics committee is, according to Dutch law, required for studies in which data are collected from the records by the treating physician.

### Outcome measures

INR and dosage of VKAs were determined on the last visit before and the first visit after start of statin use to assess an immediate anticoagulant effect. We determined the immediate difference in INR as this reflects the amount of coagulation at a particular time point. The immediate change in dosage of phenprocoumon or acenocoumarol after first statin use was expected to decrease if statins would increase the INR as VKA dosage is based on INR results. To study the long-term effect of statins on coagulation, the VKA dosage 6 and 12 weeks after statin initiation was compared with the last dosage before statin initiation. If no INR was available at these exact dates, the INR closest to the specific date of statin initiation was chosen. The differences in INR and dosage were also expressed in percentages.

### Statistical analysis

Data for continuous variables are expressed as means with standard deviations (SDs), and categorical data are expressed as numbers with percentages. In this study, patients are compared with themselves (cross-over analysis). Therefore, the mean difference in INR and VKA dosage with 95 % CI was estimated by means of linear regression and was adjusted for study centre. The reference category in all analyses was the INR and VKA dosage at the last known date before first statin use*.* All statistical analyses were performed with R version 3.1.1.

## Results

### Clinical characteristics

Thirty-two thousand, two hundred ninety patients used VKAs between 2009 and 2013, of which 12,074 used phenprocoumon and 20,216 used acenocoumarol. Of these VKA users, 1273 and 792 initiated a statin during VKA treatment, respectively. Statin initiators who were not admitted to a hospital and did not initiate or stop drugs that interact with VKAs during the study period were included for the analysis, resulting in 435 and 303 statin initiators on phenprocoumon and acenocoumarol, respectively.

The mean age of the patients was 70 years (± standard deviation 10) when starting statin therapy (Table 1). The most common indication for VKAs was atrial fibrillation (*n* = 537, 73 %) and 438 patients (59 %) were male. Simvastatin was the most initiated statin (*n* = 516, 70 %), while rosuvastatin was not initiated among phenprocoumon users in this sample. One patient started fluvastatin therapy among the phenprocoumon as well as among acenocoumarol users. Clinical characteristics were similar in acenocoumarol and phenprocoumon users and all patients kept the same INR target range during the study period.

**Table 1:** Clinical characteristics

	Phenprocoumon	Acenocoumarol
Patients	435	303
Age	70 (10)	69 (11)
Men	262 (60)	176 (58)
Indication phenprocoumon treatment^a^		
Atrial fibrillation	337 (78)	200 (66)
Venous thrombosis	53 (12)	34 (11)
Mechanical heart valves	13 (3)	24 (8)
Vascular surgery	13 (3)	10 (3)
Ischemic heart disease	20 (5)	23 (8)
Other	12 (3)	1 (0)
Target range INR		
2.5–3.5	404 (93)	242 (80)
3.0–4.0	31 (7)	61 (20)
Type of statin used		
Simvastatin	310 (71)	206 (68)
Atorvastatin	60 (14)	51 (17)
Pravastatin	64 (15)	17 (6)
Rosuvastatin	0 (0)	28 (9)
Fluvastatin	1 (0)	1 (0)

Continuous variables denoted as mean (standard deviation), categorical variables as number (%)

^a^Numbers do not add up to 100 % as patients may have multiple indications for VKA treatment

### Immediate INR and dosage change

Table 2 shows the INRs and mean VKA dose immediately after starting statin treatment in phenprocoumon and acenocoumarol users. After starting statin treatment, patients had an appointment at the anticoagulation clinic after on average 1 week. The immediate average INR increase in phenprocoumon users was 0.10 (95 % CI 0.04 to 0.17) or 6 % (95 % CI 3 to 8 %). In acenocoumarol users, no immediate change in INR was observed (INR 0.02 [95 % CI −0.10 to 0.14] increased). The mean difference of daily dosage of phenprocoumon users was 0.02 mg per day (95 % CI 0.00 to 0.03) lower and for acenocoumarol users 0.04 mg per day (95 % CI 0.01 to 0.07) lower. Stratification by statin type showed that both INR changes and dose changes were similar between the different types of statins.

**Table 2:** Immediate effect on INR and dosage after initiation of statin in VKA users

		Mean INR	(95 % CI)	Mean diff. INR	(95 % CI)	Percentage difference	(95 % CI)		Mean dosage (mg/day)	(95 % CI)	Mean diff. (mg/day)	(95 % CI)	Percentage difference	(95 % CI)
Phenprocoumon														
Any statin														
Last date before start statin use	*n* = 435	2.96	(2.72 to 3.20)	Reference		Reference		*n* = 435	1.91	(1.58 to 2.24)	Reference		Reference	
First date after start statin use	*n* = 435	3.15	(2.86 to 3.43)	0.10	(0.04 to 0.17)	6	(3 to 8)	*n* = 435	1.88	(1.55 to 2.21)	−0.02	(−0.03 to 0.00)	−1	(−1 to 0)
Simvastatin														
Last date before start statin use	*n* = 310	3.03	(2.76 to 3.31)	Reference		Reference		*n* = 310	2.10	(1.70 to 2.49)	Reference		Reference	
First date after start statin use	*n* = 310	3.18	(2.84 to 3.53)	0.13	(0.05 to 0.22)	6	(4 to 9)	*n* = 310	2.06	(1.68 to 2.45)	−0.02	(−0.03 to −0.01)	−1	(−1 to −1)
Atorvastatin														
Last date before start statin use	*n* = 60	2.63	(1.85 to 3.41)	Reference		Reference		*n* = 60	1.29	(0.33 to 2.26)	Reference		Reference	
First date after start statin use	*n* = 60	2.72	(2.02 to 3.42)	−0.01	(−0.17 to 0.16)	3	(−4 to 9)	*n* = 60	1.29	(0.35 to 2.23)	−0.01	(−0.03 to 0.01)	0	(−1 to 1)
Pravastatin														
Last date before start statin use	*n* = 64	2.83	(2.69 to 2.98)	Reference		Reference		*n* = 64	2.10	(1.90 to 2.30)	Reference		Reference	
First date after start statin use	*n* = 64	2.89	(2.73 to 3.05)	0.06	(−0.10 to 0.21)	4	(−2 to 9)	*n* = 64	2.10	(1.89 to 2.30)	0.00	(−0.02 to 0.01)	0	(−1 to 0)
Acenocoumarol														
Any statin														
Last date before start statin use	*n* = 303	2.91	(2.80 to 3.02)	Reference		Reference		*n* = 303	2.66	(2.45 to 2.86)	Reference		Reference	
First date after start statin use	*n* = 303	3.04	(2.88 to 3.20)	0.02	(−0.10 to 0.14)	4	(0 to 9)	*n* = 303	2.63	(2.42 to 2.83)	−0.04	(−0.07 to −0.01)	−1	(−3 to 0)
Simvastatin														
Last date before start statin use	*n* = 206	2.92	(2.78 to 3.05)	Reference		Reference		*n* = 203	2.69	(2.46 to 2.93)	Reference		Reference	
First date after start statin use	*n* = 206	3.06	(2.87 to 3.24)	0.02	(−0.11 to 0.17)	4	(0 to 9)	*n* = 203	2.66	(2.42 to 2.90)	−0.04	(−0.08 to −0.01)	−2	(−3 to 0)
Atorvastatin														
Last date before start statin use	*n* = 51	2.92	(2.62 to 3.21)	Reference		Reference		*n* = 51	2.71	(2.12 to 3.30)	Reference		Reference	
First date after start statin use	*n* = 51	2.94	(2.51 to 3.37)	−0.01	(−0.34 to 0.32)	6	(−7 to 19)	n = 51	2.68	(0.35 to 2.23)	−0.02	(−0.06 to 0.03)	−2	(−3 to 0)
Pravastatin														
Last date before start statin use	*n* = 17	2.89	(2.54 to 3.24)	Reference		Reference		*n* = 17	2.04	(1.54 to 2.53)	Reference		Reference	
First date after start statin use	*n* = 17	3.09	(2.46 to 3.73)	0.17	(−0.37 to 0.70)	8	(−12 to 28)	*n* = 17	2.00	(1.54 to 2.45)	−0.06	(−0.33 to 0.21)	3	(−19 to 26)
Rosuvastatin														
Last date before start statin use	*n* = 28	3.15	(2.90 to 3.40)	Reference		Reference		*n* = 28	3.04	(2.49 to 3.60)	Reference		Reference	
First date after start statin use	*n* = 28	3.15	(2.88 to 3.43)	0.01	(−0.30 to 0.31)	3	(−7 to 13)	*n* = 28	3.02	(2.47 to 2.57)	−0.02	(−0.04 to 0.00)	−1	(−1 to 0)

### Long-term dosage change

Table 3 shows the long-term change in VKA dosage after initiating statin therapy in acenocoumarol and phenprocoumon users. The mean difference in daily dosage of phenprocoumon users was 0.03 mg/day (95 % CI 0.01 to 0.05) lower after 6 weeks and 0.07 mg/day (95 % CI 0.04 to 0.09) lower after 12 weeks. The mean difference in daily dosage of acenocoumarol users was 0.10 mg/day (95 % CI 0.03 to 0.16) lower after 6 weeks and 0.11 mg/day (95 % CI 0.04 to 0.18) lower after 12 weeks. After analyses were stratified by statin type, it appeared that a stronger decrease of VKA dosage was present in simvastatin (among acenocoumarol and phenprocoumon users) and rosuvastatin users (among acenocoumarol users) as compared with the other types of statins.

**Table 3:** Long term effect of statin initiation on VKA dosage

		Mean dosage (mg/day)	(95 % CI)	Mean difference dosage (mg/day)	(95 % CI)	Mean difference dosage percentage	(95 % CI)
Phenprocoumon							
Any statin							
Last date before start statin use	*n* = 435	1.91	(1.58 to 2.24)	Reference		Reference	
6 weeks after start statin use	*n* = 434	1.85	(1.52 to 2.17)	−0.03	(−0.05 to −0.01)	−1	(−2 to 0)
12 weeks after start statin use	*n* = 408	1.81	(1.49 to 2.13)	−0.07	(−0.09 to −0.04)	−3	(−4 to −1)
Simvastatin							
Last date before start statin use	*n* = 310	2.10	(1.70 to 2.49)	Reference		Reference	
6 weeks after start statin use	*n* = 309	2.02	(1.63 to 2.40)	−0.04	(−0.07 to −0.02)	−2	(−3 to 0)
12 weeks after start statin use	*n* = 388	1.97	(1.60 to 2.34)	−0.08	(−0.11 to −0.04)	−3	(−4 to −1)
Atorvastatin							
Last date before start statin use	*n* = 60	1.29	(0.33 to 2.26)	Reference		Reference	
6 weeks after start statin use	*n* = 60	1.29	(0.33 to 2.25)	0.00	(−0.03 to 0.03)	0	(−1 to 1)
12 weeks after start statin use	*n* = 57	1.28	(0.31 to 2.26)	−0.02	(−0.07 to 0.02)	−1	(−3 to 0)
Pravastatin							
Last date before start statin use	*n* = 64	2.10	(1.90 to 2.30)	Reference		Reference	
6 weeks after start statin use	*n* = 64	2.02	(1.88 to 2.30)	−0.01	(−0.04 to 0.01)	−1	(−2 to 0)
12 weeks after start statin use	*n* = 62	2.06	(1.85 to 2.27)	−0.05	(−0.09 to −0.02)	−3	(−5 to −1)
Acenocoumarol							
Any statin							
Last date before start statin use	*n* = 303	2.66	(2.45 to 2.86)	Reference		Reference	
6 weeks after start statin use	*n* = 303	2.64	(2.44 to 2.84)	−0.10	(−0.16 to −0.03)	−2	(−4 to 1)
12 weeks after start statin use	*n* = 300	2.63	(2.42 to 2.84)	−0.11	(−0.18 to −0.04)	−2	(−5 to 1)
Simvastatin							
Last date before start statin use	*n* = 206	2.70	(2.46 to 2.93)	Reference		Reference	
6 weeks after start statin use	*n* = 206	2.62	(2.39 to 2.86)	−0.14	(−0.22 to −0.06)	−4	(−6 to 0)
12 weeks after start statin use	*n* = 204	2.59	(2.35 to 2.84)	−0.17	(−0.26 to −0.07)	−4	(−7 to −1)
Atorvastatin							
Last date before start statin use	*n* = 51	2.71	(2.12 to 3.30)	Reference		Reference	
6 weeks after start statin use	*n* = 51	2.99	(2.44 to 3.54)	0.02	(−0.11 to 0.16)	2	(−5 to 9)
12 weeks after start statin use	*n* = 50	3.20	(2.53 to 3.86)	0.08	(−0.08 to 0.24)	4	(−5 to 12)
Pravastatin							
Last date before start statin use	*n* = 17	2.04	(1.54 to 2.53)	Reference		Reference	
6 weeks after start statin use	*n* = 17	2.05	(1.60 to 2.51)	0.02	(−0.26 to 0.31)	9	(−16 to 35)
12 weeks after start statin use	*n* = 17	2.04	(1.59 to 2.51)	0.02	(−0.30 to 0.33)	10	(−18 to 39)
Rosuvastatin							
Last date before start statin use	*n* = 28	3.04	(2.49 to 3.60)	Reference		Reference	
6 weeks after start statin use	*n* = 28	2.97	(2.45 to 3.50)	−0.07	(−0.12 to −0.02)	−1	(−3 to 0)
12 weeks after start statin use	*n* = 28	2.94	(2.43 to 3.44)	−0.10	(−0.20 to −0.01)	−2	(−6 to 0)

## Discussion

The current study investigated the effect on anticoagulant properties within 738 patients on VKA therapy who initiated statins. Results on immediate INR differences showed that the INR increased slightly in phenprocoumon users but not in acenocoumarol users. The effect of initiating statin treatment on the INR was also investigated in two randomised studies in healthy volunteers [2, 6]. The study from Yu Cu et al. showed an INR increase of 0.16 9 days after rosuvastatin initiation [2], and the study by Jindal et al. found no INR difference 7 days after rosuvastatin initiation [6]. The results of our study confirm the results of these trials where the immediate INR increase was also close to null. In addition, the VKA dosage decreased in both phenprocoumon and acenocoumarol users which became apparent for both VKAs after 6 to 12 weeks. The results showed that initiating statin treatment is associated with a decrease of VKA dosage after 6 and 12 weeks, which suggests that statins interact with VKAs or have anticoagulant properties.

A potential explanation for the decrease of VKA dosage in statin users is confounding. However, we did take confounding into account, as we compared patients with themselves in which, confounding by fixed (constant) characteristics (e.g. diabetes mellitus, hypertension and genetics) is eliminated. At the time that this study was conducted, INR target ranges in the Netherlands were higher as compared to international guidelines. Because patients are compared with themselves and the INR target range stayed the same during the study period, INR target range could not have confounded our results. However, transient risk factors can introduce (non-fixed) confounding [10]. For example, one non-fixed confounding factor is the initiation of concomitant medication or experiencing a cardiovascular event (e.g., a myocardial infarction). To avoid this type of non-fixed confounding, we excluded all patients who started or stopped medications that interact with VKAs during the study period or were admitted to the hospital. Another non-fixed confounding factor is an acute transient disease, for example fever [11]. However, such a transient disease is unlikely to explain the long-term (6–12 weeks) effect that statins had on the VKA dosage in our study. A further possibility for the decrease in VKA dosage that we found after statin was initiated is that statins interact with VKAs. Acenocoumarol and phenprocoumon are racemic mixtures where the enantiomer largely responsible for the anticoagulant effect are metabolised by CYP3A4 and CYP2C9 [12]. Stratification by type of statin showed that rosuvastatin and simvastatin were associated with the strongest decrease in VKA dosage. Rosuvastatin is only 10 % metabolised by CYP2C9, while simvastatin is metabolised by CYP3A4 [13]. The dosage decrease after initiation of rosuvastatin, which is hardly metabolised by CYP2C9, suggests that our results are not likely to be explained by drug-drug interactions. In addition, differences in lipophilicity of statins are also unlikely to account for the differences found between statins as rosuvastatin is hydrophilic while simvastatin is lipophilic. A potential other explanation is that statins do reduce coagulation, which was suggested by Sahebkar et al. because D-dimer levels decreased after 3 months of statin therapy and because D-dimer levels are markers of coagulation [14]. To get more insight whether simvastatin and rosuvastatin have anticoagulant properties, a next step would be to investigate the effect of these statins on coagulation in patients not on VKAs.

Though the current study is of etiological interest as it gives a lead why statins might be able to decrease venous thrombosis risk, its clinical effect appears to be minimal: INRs did not increase immediately and only marginally, and the VKA dose reduction was also minimal.

A potential limitation of our study is that co-medication was self-reported and the only statin reported by the pharmacy to the anticoagulation clinics was rosuvastatin. Consequently, there may be discrepancies between the medication records of the anticoagulation clinics and what the patients used. As patients were compared with themselves, we expect that this has not influenced the results. An additional limitation is that we excluded patients who were hospitalised between the INR measurement before and after statin initiation. We did this because the assumption of the study, that there are no other environmental changes present that can affect VKA dosage and/or INR in the patient except that the patient started with statin, is otherwise not held. For that reason, we could have missed patients of more ‘dramatic’ changes of anticoagulation, like patients with a major bleed. Furthermore, pharmacokinetics of the two studied VKAs do differ, for example phenprocoumon has a longer half-life as compared with acenocoumarol [12]. However, differences in pharmacokinetics of the VKAs tested are unlikely to have contributed to the statin results found in this study as results were similar in both acenocoumarol and phenprocoumon users. Another limitation is that we assumed that patients are compliant to their statin therapy. It is likely that not all patients were fully compliant as previous studies showed an average adherence to statins of 71–77 % [15]. Our results could therefore be diluted and the effects on VKA dosage are likely to be stronger if we could have taken statin adherence into close account. A final limitation of our study is that the dosage of statins was not registered in the electronic system. Therefore, no analyses could be performed that took the dosage of statin into account.

In conclusion, we found that statin treatment was associated with a minor although statistically significant decrease in VKA dosage in both phenprocoumon and acenocoumarol users, which suggests that statins may have anticoagulant properties.
